# Evaluating the Prevalence and correlates of high stress and low resilience among Educators

**DOI:** 10.1192/j.eurpsy.2025.810

**Published:** 2025-08-26

**Authors:** A. Belinda, R. D. L. Dias, Y. Wei, V. I. O. Agyapong

**Affiliations:** 1 University of Alberta, Edmonton; 2 Dalhousie University, Halifax, Canada

## Abstract

**Introduction:**

High-stress levels can be problematic for teachers and indirectly affect students. Knowledge about the prevalence and predictors of high-stress and low resilience will provide information about the extent of the problem among teachers in Canada.

**Objectives:**

To examine the prevalence and correlates of perceived stress and low resilience among Alberta, Nova Scotia, Newfoundland and Labrador teachers.

**Methods:**

This is a cross-sectional study. Participants self-subscribed to the Wellness4Teachers text-messaging program and completed the online survey on enrollment. Data collection occurred from September 2022 to August 2023. Resilience and stress were respectively assessed using the Brief Resilience Scale (BRS) and the Perceived Stress Scale (PSS-10). Data was analyzed with SPSS version 28.

**Results:**

A total of 1912 teachers subscribed to the Wellness4Teachers program, and 810 completed the baseline survey, yielding a response rate of 42.40%. The prevalence of high stress and low resilience were respectively 26.3%, and 40.1%. Participants with low resilience were 3.10 times more likely to experience high-stress symptoms than those with normal to high resilience (OR = 3.10; 95% CI: 2.18–4.41). Conversely, participants who reported high stress were 3.13 times more likely to have low resilience than those with low to moderate stress (OR = 3.13; 95% CI: 2.20–4.44).

**Conclusions:**

Our study findings infer there’s an incidence of high levels of stress and low resilience among teachers in the three Canadian provinces. Governments and policymakers in the education field should integrate stress management and resilient building strategies into teachers’ ongoing professional development programs to help prevent and address high stress.

**Disclosure of Interest:**

None Declared

